# Using Exploratory Trials to Identify Relevant Contexts and Mechanisms in Complex Electronic Health Interventions: Evaluating the Electronic Patient-Reported Outcome Tool

**DOI:** 10.2196/11950

**Published:** 2019-02-27

**Authors:** Carolyn Steele Gray, Janelle Gravesande, Parminder Kaur Hans, Jason X Nie, Sarah Sharpe, Mayura Loganathan, Renee Lyons, Cheryl Cott

**Affiliations:** 1 Bridgepoint Collaboratory for Research and Innovation Lunenfeld-Tanenbaum Research Institute Sinai Health System Toronto, ON Canada; 2 Institute of Health Policy, Management and Evaluation Dalla Lana School of Public Health University of Toronto Toronto, ON Canada; 3 School of Rehabilitation McMaster University Hamilton, ON Canada; 4 Corporate Services Trillium Health Partners Toronto, ON Canada; 5 Institute for Better Health Trillium Health Partners Toronto, ON Canada; 6 QoC Health Inc Toronto, ON Canada; 7 Ray D Wolfe Department of Family Medicine Mount Sinai Hospital Toronto, ON Canada; 8 Department of Family and Community Medicine Faculty of Medicine University of Toronto Toronto, ON Canada; 9 Department of Physical Therapy University of Toronto Toronto, ON Canada

**Keywords:** eHealth, mHealth, multiple chronic conditions, clinical trial, phase III, health care evaluation mechanisms, quantitative evaluation, qualitative evaluation, narrative analysis

## Abstract

**Background:**

Designing appropriate studies for evaluating complex interventions, such as electronic health solutions to support integrated care, remains a methodological challenge. With the many moving parts of complex interventions, it is not always clear how program activities are connected to anticipated and unanticipated outcomes. Exploratory trials can be used to uncover determinants (or mechanisms) to inform content theory that underpins complex interventions before designing a full evaluation plan.

**Objective:**

A multimethod exploratory trial of the electronic patient-reported outcome (ePRO) tool was conducted to uncover contexts, processes and outcome variables, and the mechanisms that link these variables before full-scale evaluation. ePRO is a mobile app and portal designed to support goal-oriented care in interdisciplinary primary health care practices (clinical-level integration). This paper offers evaluation findings and methodological insight on how to use exploratory trial data to identify relevant context, process, and outcome variables, as well as central (necessary to achieving outcomes) versus peripheral (less critical and potentially context dependent) mechanisms at play.

**Methods:**

The 4-month trial was conducted in 2 primary health care practices in Toronto, Canada. The patients were randomized into control and intervention groups and compared pre and post on quality of life and activation outcome measures. Semistructured interviews were conducted with providers and patients in the intervention group. Narrative analysis was used to uncover dominant mechanisms that inform the intervention’s content theory (how context and process variables are linked to outcomes).

**Results:**

Overall, 7 providers, 1 administrator, and 16 patients (7-control, 9-intervention) participated in the study. This study uncovered many complex and nuanced context, process, and outcome variables at play in the intervention. Narrative analysis of patient and provider interviews revealed dominant story lines that help to tease apart central and peripheral mechanisms driving the intervention. Provider and patient story lines centered around fitting the new intervention into everyday work and life of patients and providers and meaningfulness of the intervention. These themes were moderated by patient-provider relationships going into and throughout the intervention, their comfort with technology, and the research process.

**Conclusions:**

Identifying dominant story lines using narrative analysis helps to identify the most relevant context and process variables likely to influence study outcomes. Normalization process theory emerges as a useful theory to uncover underlying mechanisms because of its emphasis on the social production and normalization of technological, processual, and social aspects of work; all found to be critical to our intervention. The number of complex, overlapping influencing variables suggests that complex interventions such as ePRO require us to pay careful attention to central versus peripheral mechanisms that will influence study outcomes. The narrative methods presented here are shown to be useful in uncovering these mechanisms and help to guide subsequent larger evaluation studies.

## Introduction

### Background

Designing rigorous and appropriate evaluation studies for complex interventions, such as electronic health (eHealth) solutions to support integrated care, remains a methodological challenge. Although it is difficult to draw the line between simple and complex, complex interventions tend to include multiple interacting components that might occur across multiple individuals, teams, or organizations. These interventions can have numerous and variable expected and unexpected outcomes, which occur through multiple potentially challenging behavior changes by those delivering and receiving interventions. Contributing to complexity is the number of interacting components and degree of fidelity—degree to which a program is delivered as intended [1]—required of the intervention [2].

Interventions involving eHealth solutions are among those complex examples that make evaluation challenging. eHealth can be broadly defined as information and communication technologies used as part of health service delivery [3]. eHealth and mobile technology adoption is often viewed as a prime example of a complex health intervention, given the interaction of individual, contextual, and technological variables [4-6]. As such, evaluation methods used for complex interventions are recommended to assess eHealth interventions.

The Medical Research Council’s framework on the evaluation of complex interventions includes attention to the stages of implementation from development to full-scale implementation [2,7]. Similarly, Parry et al [8] recommend adopting a 3-phase approach to evaluation of new complex models of care. These phased approaches seek to build an understanding of the intervention to inform evaluation designs. Common among the approaches are attention to the context in which interventions are implemented and the mechanisms (or content theory), which suggest how the intervention will lead to expected outcomes [7,8]. Moreover, common is a view to an end point of a randomized control design, which persists as the gold standard within the hierarchy of evidence [9,10].

Exploratory trials are recommended as a means to pilot test key components of a full trial including the control (or comparative) group, the appropriate sample size, and suitable outcome measures relevant to patients and at the system level (ie, economic measures) [2,11]. Exploratory trials can also be used to refine content theory as well as for exploring the implementation of the model in terms of the satisfaction, experience, and learning among users (execution theory) [8]. However, a recent systematic review of the complex intervention evaluation literature found there is often insufficient reporting of detail in these evaluations, particularly around context of the intervention itself [12]. As such, there is a lack of methodological guidance in conducting these trials and using them to inform larger-scale evaluations.

This paper presents findings from our exploratory trial of the Health System Performance Research Network-Bridgepoint *electronic Patient-Reported Outcomes* mobile device and portal system—hereafter referred to as the electronic patient-reported outcome (ePRO) tool—designed to support goal-oriented care delivery in primary care settings. We adopted a developmental evaluation approach collecting quantitative and qualitative data to support the appraisal of the tool. In addition to testing the trial design, this exploratory trial sought to determine how we draw on multiple data sources to gain insight into the relevant contexts, processes and outcomes, and the mechanisms that connect these variables. This paper offers study findings as well as methodological insight with regard to how we answer 2 questions:

1. What are the contexts, processes, and outcomes most relevant to the ePRO intervention?

2. What are the central (critical to achieving outcomes) versus peripheral (less critical and potentially context dependent) mechanisms that underpin the content theory of the ePRO intervention?

### The Intervention: The Electronic Patient-Reported Outcome Tool

The ePRO tool includes 2 features: (1) My Goal Tracker and (2) Health Status Scales and Outcome Measures. See Multimedia Appendix 1 for screen shots of the portal and mobile app and Steele Gray et al [13] for a detailed overview of ePRO.

**Table 1:** Goal attainment scale monitoring.

Score	Goal achievement
+2	Much better than expected
+1	Better than expected
0	Goal (expected level specified by patient and, or caregiver and provider)
–1	Less than expected
–2	Much less than expected

**Table 2:** Monitoring protocols.

Symptom or outcome	Health status scales and outcome measures
Depression	Patient Health Questionnaire 9-item depression questionnaire [15]
Anxiety	Generalized Anxiety Disorder 7-item anxiety questionnaire [16]
Global health	PROMIS^a^ Global Health Scale^b^
Pain management	PROMIS Pain Interference Scale^b^
Mobility	Improved Health Assessment Questionnaire^a^

^a^PROMIS: Patient-Reported Outcomes Measurement Information System.

^b^See [17-20] validation of PROMIS tools and relevance to primary care.

#### Feature #1: My Goal Tracker

*My Goal Tracker* allows patients (their caregivers if interested) and providers to collaboratively create goal-oriented patient care plans. Once a goal is added to the patients’ care plan, their mobile app prompts them to report on outcomes related to that goal on their mobile device (specifically on a smartphone). The ePRO tool uses goal-attainment scaling (depicted in Table 1) to capture standardized outcome measures across diverse patient groups, standardize goal attainment measures, and address the challenge of writing multiple goals [6]. Customizable monitoring questions can be added using question templates. Patients can include comments at each monitoring period to provide context and detail needed to understand why goals are achieved or are not achieved.

#### Feature #2: Health Status Scales and Outcome Measures

The Health Status Scales are intended to help patients, their caregivers, and providers track and monitor symptoms and outcomes identified as important by patients with complex care needs [14]. This type of monitoring is also helpful for patients not yet ready to goal set, as it provides a starting point to the self-monitoring process. Similar to the My Goal Tracker feature, patients will be prompted when symptom reporting is due. Table 2 identifies monitoring protocols that can be added to patient health journals.

## Methods

### Evaluation Framework

As recommended for exploratory trials and complex interventions, we sought to capture context, process, and outcome measures to provide early evidence of effectiveness (or ineffectiveness) of the tool and to identify likely *mechanisms of action* to build on what was learned in piloting [7,11]. Consistent with the developmental evaluation approach, informed by our overarching user-centered design methods, data from this study are also used to inform design changes to the technology—see prior publications on development and usability testing [21,22]. Table 3 summarizes outcome, process, and outcome variables of interest to our study, and the tools and methods used to capture data.

#### Outcome Measures

Patient and provider level outcomes were collected as part of this study. The primary patient outcome measures included the following: (1) Quality of life, measured using the Assessment of Quality of Life Scale (AQoL-4D) [23] and (2) self-management, measured using the 13-item Patient Activation Measure (PAM) [24]. Patient experience measures were collected using a modified version of the Patient Assessment of Chronic Illness Care (PACIC) tool, a 20-item measure of patient centeredness [25]. Provider-level effectiveness was captured using provider interviews informed by the Assessment of Chronic Illness Care tool (the provider partner assessment to the PACIC), which has been used to help health care teams improve care delivered to patients with chronic illness [26].

#### Process Measures

The Post-Study System Usability Questionnaire (PSSUQ) was used to assess experience and usability of the tool. The PSSUQ is a 19-item usability questionnaire comprising 3 subscales (system usefulness, information quality, and interface quality) [27]. The PSSUQ has demonstrated reliability and validity [28] and has been used to assess satisfaction and experience with similar mobile health technologies [27]. Postintervention patient and provider interviews were used to capture additional information regarding user experience and probed tool impact on provider workflow. Although ethnography and observation methods can be used to assess these process measures [29,30], these approaches were not feasible for this study. Instead, we used targeted probes in the interviews to capture data.

#### Context Measures

Context measures are captured at the patient, provider, organizational, and system level. At the patient and provider level, demographic and characteristic information such as age, gender, chronic illness profile, socioeconomic status, and information technology (IT) skills are collected. These contextual factors have been found to impact the adoption and implementation of eHealth tools [31]. At the organization and system levels, we used postimplementation interviews with providers and clinic managers to identify barriers and facilitators to ePRO adoption. Factors such as appropriate supportive resources (ie, IT support), logistical issues (ie, integration of the tool into provider workflows), appropriate training and time to learn new systems, and organizational-level support have been found to be pivotal in adopting new eHealth systems [31-33]. System level contextual issues such as noncentralized systems, lack of standardization of data systems, legal requirements, financial incentives (or disincentives) have also been found to impact eHealth adoption [32].

### Study Design

We conducted a 4-month trial in 2 Family Health Teams (FHTs) in Toronto. To study outcomes, we randomized patients into either control or intervention arms of the study at the 2 sites. To explore process and context measures, we adopted a case study approach, which is appropriate for complex interventions given the need to explore phenomena in their natural settings [34]. These types of naturalistic designs are recommended when evaluating telehealth and eHealth interventions [4].

### Setting and Population

FHTs are primary health care models with an interprofessional practice team including primary care clinicians, nurse practitioners, practice nurses, and other allied health staff (eg, dietitians, social workers) [35]. We aimed to recruit 30 patients per site where 15 would be randomized into intervention and the remaining would act as the control group. Patients to be included in the study were first identified through electronic medical records (EMRs) using the following eligibility criteria: (1) rostered patient to the practice, (2) 10 or more visits to the clinic within the previous 12 months, and (3) 5 or more medications. Generated EMR lists were reviewed by providers to screen for patients who they considered had complex care needs. Once a list was finalized, patients were invited to participate through recruitment letters mailed to their homes.

### Data Collection

Table 3 summarizes our evaluation framework including outcome, process, and context measures collected at the patient, provider, and system levels. As noted in the table, interviews with intervention patients, providers, managers, and administrative staff were conducted at the end of the study. These semistructured interviews were conducted one-on-one between the participant and 1 member of the research team. The patient interviews were conducted immediately following the off-boarding session with the provider (the last appointment where they discussed goals at the end of the study) and lasted between 30 and 60 min. Providers were interviewed in their offices, interviews lasting between 30 and 60 min, within 3 months of the end of the trial.

**Table 3:** Data collection for electronic patient-reported outcome tool exploratory trial.

Concept and measurement level		Variable	Tool and method	Data collection
**Outcome**				
	Patient	Self-management	Patient Activation Measure	Baseline; 4 months
		Quality of life	Assessment of Quality of Life Scale	Baseline; 4 months
		Person-centered care delivery	Patient Assessment of Chronic Illness Care	Baseline; 4 months
	Provider	Delivering patient-centered care	Provider interviews	Postintervention
**Process**				
	Patient	Tool experience	Post-Study System Usability Questionnaire	4 months
	Patient	Tool experience	Patient interviews	Postintervention
	Provider	Tool experience	Provider interviews	Postintervention
	Organization	Provider workflows	Provider interviews	Postintervention
**Context**				
	Patient	Patient demographic and characteristics	Electronic medical record extraction Patient information sheet	Preintervention
	Provider	Provider demographic and characteristics	Provider information sheet	Preintervention
	Organization	Resources, support, and training	Provider and manager interviews	Postintervention
	System	Structure, data standards, legal requirements, and funding	Provider and manager interviews	Postintervention

Although the length of time from end of trial to interviews was a bit longer for providers, this was unavoidable because of challenges faced while booking interview times with busy clinicians. Given it was a unique experience for most providers, they were able to recall the experience and provide in-depth feedback. Only 1 provider explicitly noted some challenges in recalling the intervention.

Patients and providers were asked to tell us the story about how they used the tool over the 4 months, what (if anything) changed because they used the tool, the challenges and benefits that were experienced, and then more directed questions and probes around the usability of the tool. Providers and managers were additionally asked to reflect on enablers and barriers from a clinical and organizational perspective as they told the story of using the ePRO tool. Interviews were audio-recorded and transcribed verbatim. As is consistent with qualitative analysis methods [36], postinterview memos were written by researchers who conducted the interviews and included in the dataset to guide analysis.

### Data Analysis

Patient outcome data were analyzed by calculating overall and domain scores of the AQoL-4D (standardized scores of Independent Living, Mental Health, Relationships, Senses, and total), PACIC (Patient Activation, Delivery System Design and Decision Support, Goal Setting, Problem-solving and Contextual Counseling, Follow-up and Coordination, and total), and PAM, including changes in scores between baseline and 4-month follow-up periods within groups and between intervention and control groups. Due to the small sample size, the Wilcoxon signed-rank test was used to compare paired survey data means between pre and post for both control and intervention groups, and the Mann-Whitney test was used to compare the change in scores from pre and post between the Control and Intervention groups. Quantitative data on tool experience captured through the PSSUQ were analyzed using standard descriptive statistics across subdomains. Data were analyzed using SPSS version 25 (SPSS Inc).

Qualitative data collected through patient, provider, manager, and administrator interviews were analyzed using qualitative description [37] to first categorize and identify dominant themes in the data. Overall, 3 coauthors engaged in the iterative inductive coding of transcripts to identify codes. The coauthors double coded a subset of interviews to validate the codebook. Codes were identified at the patient, provider, organization, and system levels, as well as codes relating to the technology. Through this process, descriptive codes were categorized into thematic groupings. Once the authors agreed upon the descriptions and application of the codes, all interview transcripts were coded using NVivo 11 (QSR International). A summary of findings was shared with patient and provider participants for member checking. No suggested modifications, concerns or issues were raised by participants.

## Results

### Participants

A total of 201 patients were identified and mailed invitations (113 at site 1 and 88 at site 2). Overall, 16 patients (21 consented, 5 withdrawals) participated in the study across the 2 sites. Of them, 9 were randomized into the intervention arm and 7 to the control arm. In total, 11 of the patients were female, most of whom were in the age group categories of 55 to 64 years (n=7) or 65 to 74 years (n=5), and 9 patients reported having 3+ chronic conditions. A total of 15 patients reported either comfortable or very comfortable with a computer; 14 reported either comfortable of very comfortable with a smartphone or tablet.

The vast majority of patients who were contacted simply did not respond to our emails and follow-up phone calls. For those we were able to speak to directly, the main reasons they did not wish to participate were: (1) they were already overwhelmed with managing their health needs and did not want to add another responsibility, (2) they were not sure they had health goals they could work on, (3) they did not self-identify as having a *chronic condition* (eg, some patients did not consider hypertension or even diabetes as a chronic condition) and therefore felt they did not meet the inclusion criteria, and (4) they had concerns about using technology (they did not have much experience using technology).

A total of 6 providers participated in this study, including 2 physicians (1 of whom held a managerial role), 1 nurse practitioner, 2 registered nurses (1 a diabetes educator), 1 social worker, and 1 dietitian. A total of 6 providers reported they were comfortable with computer and tablet technologies. An administrative staff member involved in the implementation of the study was also interviewed. Interviews were conducted with all 9 intervention patients, all 6 providers (1 of whom was also a clinical lead), and the 1 member of the administrative staff. All interviews were conducted at one time.

### Electronic Patient-Reported Outcome Contexts, Processes, and Outcomes

**Figure 1:**
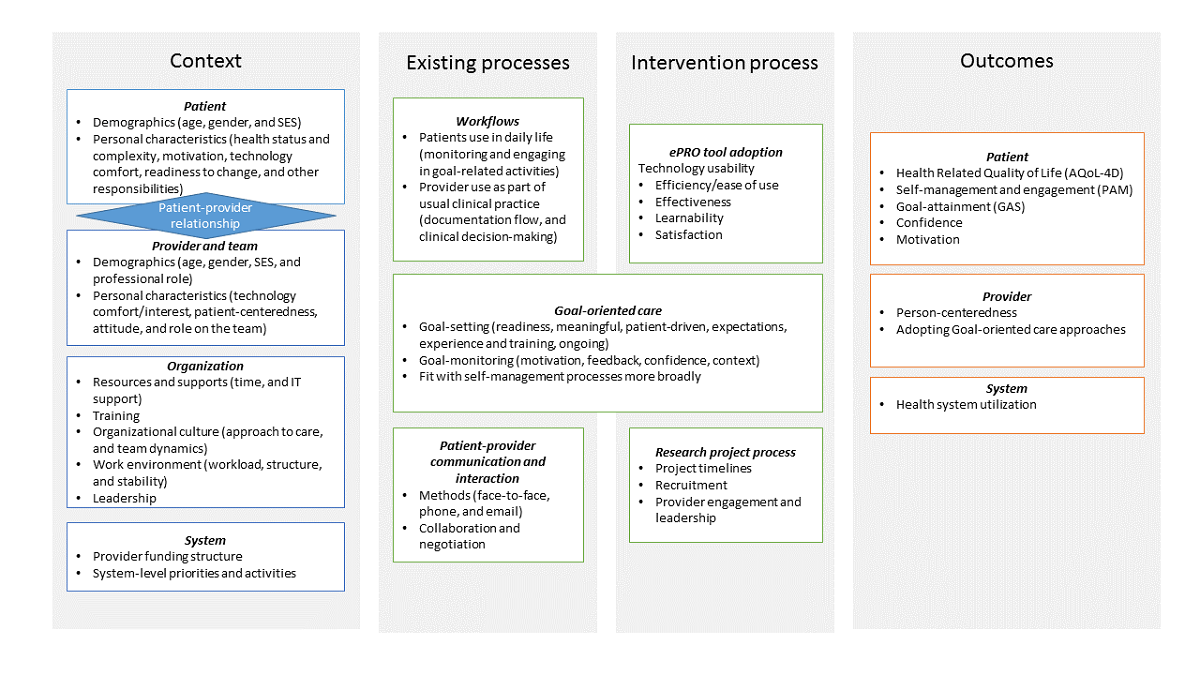
Context, process, and outcome constructs present in the electronic patient reported outcome exploratory trial. AQoL-4D: Assessment of Quality of Life Scale; GAS: goal-attainment scale; PAM: Patient Activation Measure; SES: socioeconomic status.

Quantitative and qualitative data from the exploratory trial were mapped onto a context, process, and outcome framework. Although some of this mapping came directly from our design (see variables described in Table 3), we pulled data from interviews to identify additional variables perceived as having an impact on the intervention. Variables extracted from our interviews include the following: the patient-provider relationship, patient motivation, confidence and responsibilities, provider attitudes and beliefs, organizational culture and work environment, patient use in daily life, the process and adoption of the goal-oriented care process (beyond just the use of technology), additional outcomes including patient goal-attainment, and perhaps most notably the research project process. Figure 1 offers a comprehensive descriptive list of all identified constructs.

#### Electronic Patient-Reported Outcome Context

As depicted in Figure 1, contextual variables at patient, provider, organization, and system level were all found to play a likely role in the ePRO intervention. Not only are there multiple components to consider at each level but these levels also intersect. In particular, the patient-provider relationship was found to be a critical context for this intervention, which also drove some key mechanisms of change (described in our Content Theory section).

#### Electronic Patient-Reported Outcome Process

Process variables can be broken into existing processes as compared with new processes introduced by the ePRO intervention. Existing processes provide an indication of the types of work conducted by providers (eg, clinical work as usual) and patients. For clinicians, work as usual includes seeing patients, engaging in decision-making activities, documenting encounters, following-up, and referring as required. For patients, usual work involves engaging in their everyday lives, which often involves managing their complex health and social care needs. The intervention process introduced *new* work for both patients and providers who were asked to engage with a novel technology as part of care. Notably, although FHTs had adopted goal-oriented care as part of usual practice, this study showed that the tool introduced new ways of engaging in this approach, making it both an existing and new process. Finally, the research project process itself played an important role (see Content Theory section).

#### Electronic Patient-Reported Outcomes

Patient pre- and postsurvey results are presented in Tables 4 and 5. No statistical differences were seen for pre versus post means of overall or subscale scores of AQoL, PACIC, and PAM in both control and intervention groups, nor between control and intervention; however, the sample is not sufficiently powered to pick-up change. As exploratory trials are often used to help determine sample size for larger trials, effect size estimates were calculated using Cohen *d*. As can be noted in Table 5, the confidence interval ranges are quite large, which is unsurprising given the small sample, and make it difficult to generate meaningful effect sizes.

Interestingly, control patients had a lower reported quality of life at baseline as compared with intervention patients, who scored above the norms reported in the literature [38]. Interestingly, the SDs are smaller than those reported norms, suggesting less variability in our relatively small sample. The greater homogeneity of the sample might be connected to some of the reasons patients chose to decline the participation described above, resulting in a group that looks a bit more similar than the broader complex patient population.

**Table 4:** Pre/postsurvey means and standard deviations of control and electronic patient-reported outcome intervention groups.

Measures		Control, mean (SD)		Intervention, mean (SD)	
		Pre	Post	Pre	Post
**Assessment of Quality of Life Scale** **(standardized scores)^a^**		69.05 (17.23)^b^	65.48 (17.56)^b^	83.33 (5.38)^c^	84.57 (11.96)^c^
	Independent living	74.60 (33.16)^b^	77.78 (22.22)^b^	95.06 (8.07)^c^	92.59 (18.43)^c^
	Relationships	63.49 (25.43)^b^	61.90 (30.67)^b^	83.95 (12.56)^c^	85.19 (11.11)^c^
	Mental health	55.56 (18.14)^b^	44.44 (22.22)^b^	65.43 (17.07)^c^	65.43 (21.11)^c^
	Senses	82.54 (24.73)^b^	77.78 (21.28)^b^	88.89 (9.62)^c^	95.06 (8.07)^c^
**Patient Assessment of Chronic Illness Care^d^**		3.25 (0.88)^b^	2.57 (1.31)^e^	3.61 (0.98)^c^	3.39 (1.16)^c^
	Patient activation	3.33 (1.00)^b^	2.87 (1.58)^e^	4.19 (0.88)^c^	3.81 (1.31)^c^
	Delivery system Design and decision support	2.86 (0.96)^b^	2.50 (1.36)^e^	4.11 (0.90)^c^	3.67 (1.19)^c^
	Goal setting	2.97 (1.15)^b^	2.29 (1.26)^e^	3.31 (1.30)^c^	3.22 (1.20)^c^
	Problem-solving and contextual counseling	3.50 (1.36)^b^	2.85 (1.75)^e^	3.78 (1.16)^c^	3.44 (1.35)^c^
	Follow-up and coordination	3.49 (0.81)^b^	2.52 (1.33)^e^	3.16 (1.13)^c^	3.09 (1.26)^c^
Patient Activation Measure^d^		55.91 (10.15)^b^	54.96 (22.34)^f^	68.90 (15.72)^c^	68.15 (17.16)^c^
**Post-Study System Usability^g^** **Questionnaire^a^**		—^h^	—	—	3.83 (1.84)^c^
	System usefulness	—	—	—	3.78 (2.24)^c^
	Interface quality	—	—	—	3.46 (1.95)^c^
	Information quality	—	—	—	3.92 (1.51)^c^

^a^High scores indicate lower functioning.

^b^n=7.

^c^n=9.

^d^High scores indicate higher functioning.

^e^n=5.

^f^n=6.

^g^PSSUQ scores were only collected post study for intervention patients who used the technology.

^h^Not applicable.

**Table 5:** Effect size and change scores for control and electronic patient-reported outcome intervention groups.

Measures		Control			Intervention			Mann Whitney test (*P* value)
		Cohen *d*	95% CI of difference	*P* value	Cohen *d*	95% CI of difference	*P* value	
**Assessment of Quality of Life Scale** **(standardized scores)^a^**		−0.23	−10.86 to 18.01	0.31	0.12	−9.37 to 6.9	0.48	0.21
	Independent living	0.17	−20.69 to 14.34	0.71	−0.12	−13.95 to 18.89	>.99	0.54
	Relationships	−0.04	−34.71 to 37.88	0.68	0.07	−14.36 to 11.89	0.89	0.76
	Mental health	−0.36	−17.34 to 39.56	0.48	0	−12.08 to 12.08	0.89	0.84
	Senses	−0.19	−18.11 to 27.63	0.5	0.63	−13.7 to 1.36	0.1	0.09
**Patient Assessment of Chronic Illness Care^b^**		−0.59	−0.38 to 1.09	0.23	−0.23	−0.52 to 0.96	0.77	0.52
	Patient activation	−0.24	−1.7 to 2.5	0.47	−0.34	−0.46 to 1.2	0.2	0.9
	Delivery system design and decision support	0.02	−1.9 to 1.83	0.89	−0.34	−0.56 to 1.45	0.39	0.7
	Goal setting	−0.29	−0.63 to 1.01	0.59	−0.07	−0.95 to 1.13	0.95	0.7
	Problem-solving and contextual counseling	−0.25	−1.2 to 1.8	0.58	−0.43	−0.29 to 0.93	0.26	0.9
	Follow-up and coordination	−1.09	−0.1 to 1.54	0.07	−0.05	−0.92 to 1.05	0.91	0.52
Patient Activation Measure^b^		−0.15	−8.08 to 10.89	0.89	−0.04	−12.07 to 13.56	0.57	0.86

^a^High scores indicate lower functioning.

^b^High scores indicate higher functioning.

Qualitative interviews point to additional outcomes beyond those anticipated in the original design. Patients reported gaining confidence and an increased motivation to engage in the behavior change needed to help achieve goals. Patients and providers also reported attaining and exceeding goals such as regularly attending health and wellness programs, improved sleep, weight loss, and improved blood pressure. Providers reported improved person-centered care delivery by having a tool to help guide collaborative conversations and better engage in goal-oriented care as a process.

The qualitative data thus provide us with a more in-depth understanding of the impact of the intervention than the quantitative data alone. These varying pictures of impact offered by quantitative and qualitative data require us to better understand what is driving the changes we are seeing. This better understanding of *why* we observe these changes can be achieved through an exploration of the mechanisms that link contexts and processes to observed outcomes. The following section offers our analysis of the mechanisms that are likely informing these outcomes.

### Using Narrative Analysis to Uncover Central and Peripheral Mechanisms

Our findings suggest a range of potentially applicable theories and frameworks that might be influencing outcomes. Theories of patient self-management and individual behavior change are likely applicable given the intention of the ePRO tool supporting goal setting and attainment. However, identifying which is most applicable can be challenging. A recent scoping review of theories of behavior change in the social and behavioral sciences literature found 82 behavior and behavior change theories available [39], dozens of which could be applicable in this intervention. In addition, usability, organizational behavior, and technology use theories, such as the Fit between Individual, Task, and Technology [33], could also apply.

Rather than identifying all possible applicable theories, frameworks, and relevant disciplinary approaches and then engaging in concept mapping, often unfeasible in tight study timelines, we decided to draw on narrative methods. Narrative and ethnographic methods are being increasingly adopted in the evaluation of complex eHealth interventions [29,40-43], and they can help capture contextual variables as a means to *illuminate* complexities and tensions [44]. Although we did not explicitly conduct a narrative study as part of our exploratory trial, the interviews with patients, providers, and administrative staff provide us with shared stories of technology use, thereby offering the opportunity to adopt a narrative analysis approach to illuminate dominant constructs likely to be influencing our outcomes.

Using a descriptive narrative approach [45], coded transcripts were reviewed to search for common themes, and short summaries were written for each transcript. Saturation of dominant themes occurred by the fourth provider and manager interview. A summary memo was written on these themes, which was shared with coauthors for review and discussion. The same method was repeated for patient interviews; saturation of themes was similarly achieved. We present dominant themes from provider and patient narratives below and use these to update Figure 1 with a visual pathway to link contexts, processes, and outcomes.

#### Provider/Manager Narrative Themes

Overall, 2 dominant themes were found across the provider, manager and administrator transcripts reviewed: (1) research versus real-world and (2) meaningfulness. Each theme has a slightly different tone and nuance across the different interviews and was moderated by context variables.

##### Research Versus Real World

Every provider discussed how the adoption of ePRO was different in the context of research than what would be expected in real-world practice. Particularly concerning to providers was timing constraints of the research study, meaning that patients had to be identified and on-boarded in a particular window, which did not fit into the way things *usually work*. Providers reflected goal-oriented care should happen organically and take into account patient readiness and the relationship between the patient and provider. Simply put, a strict research timeline did not resonate with the iterative nature of the goal-oriented care process:

I think because health coaching or change processes is such an iterative ongoing process that in the longer term,it would sort of come in and out of being useful for me, I think. In times when someone was really willing to work on a goal and really wanted to progress, then being able to track that change would have been really helpful.

Nurse

Even though the tool had been codesigned with providers to fit their usual clinical workflow, adopting ePRO in a research environment was perceived as a new process, which was challenging in a busy clinic environment where providers experience competing demands. Among the recommendations for improvement was the suggestion to integrate the technology into the practice EMR, a tool already highly used as part of the clinical workflow of providers. The clinical leader at site 1 reflected on the tension between a busy clinic and the research process; later in their story, they suggested how better alignment to clinical priorities (context) would improve adoption:

Sometimes research studies are as they are–just a little bit clunky to fit into regular life. But it was a bit of a round peg, square hole situation [...] [Providers] were making it work into the work flow as opposed to just sort of fitting into the work flow. And so I think that they got kind of stuck at that level as opposed to kind of moving on and moving forward with the patient goal.

...there’s all these layers of things [the teams] have to do [...] So we all have to have a quality improvement plan. We all have to have matrix that we need to report on [...] there’s eventually an opportunity to say, look, [ePRO] is part of either a patient satisfaction matrix or a complexity management matrix or kind of actually making it into the work, making it what people are about, they will likely, again, be a bit more engaged and use it more.

Physician and Clinical Lead

##### Meaningfulness

The second dominant theme in the providers’ stories was about the meaningfulness of the ePRO tool to themselves, their patients, and to their organization. Providers often reflected on what they felt the role of technology should be: desiring a tool that would provide actionable information to assist with clinical decision-making, in addition to improving workflow efficiencies resulting in less work” (Nurse), meaning fewer clinical activities needed to meet patient needs. Meaningfulness to the organization was often around meeting performance metrics, as identified by the clinical leader of the FHT.

In almost all stories, providers discussed how important it was that the ePRO tool be meaningful to patients. Interestingly, for most providers, the challenges regarding fitting the tool into their practice were mitigated when they felt their patients benefitted from using ePRO:

It would definitely be adding work, I would say, because it takes up that time in the appointment. It would take up more time than just having a discussion about SMART goals. But that being said, if it’s beneficial to the patients then I’m willing to do that.

Registered Dietitian, Diabetes Educator

Some competing stories emerged regarding how patient contexts would change whether the tool was meaningful to patients or not. For instance, several providers noted that patients were not ready to goal set or had needs that were too complex to engage in goal setting, stating that it would “only work for those super motivated patients” (Registered Dietitian, Diabetes Educator), whereas other providers suggested patients were *too* capable to see benefits from the tool:

[the patient] was already quite an active 70-something year old woman, that maybe she needed less sort of pushing, motivating and prompting than maybe if she was like a smoking cessation person where it is a very hard thing to change and to do which might require a lot more checks and check-ins.

Nurse Practitioner

An interesting reflection from 1 provider (and resonated with patient stories discussed below) is how the collaborative nature of the tool increased the meaningfulness of the tool for patients and helped to motivate them toward their goals:

Now, when they’ve had their own apps or their own journals, they didn’t stick to it. And I think maybe [ePRO] made them feel more responsible in a sense. Maybe because I was involved or someone else was involved, and so they felt they had that responsibility to do it. So someone did definitely lose weight using the application just because of keeping him more mobile and active. The majority said they found it useful for sure.

Physician

#### Patient Narrative Themes

The narrative analysis of patient stories revealed, 2 dominant themes, 1 around goal reminders and progress monitoring, and the other regarding the crucial role of the provider. The expression of these themes varied in terms of whether patients were techno-savvy (identifying greater technological experience and comfort) or techno-timid (reporting less experience and comfort with technology).

##### Goal Reminder and Progress Monitoring

Both patient groups talked about how useful the tool was as a reminder and as a way to monitor their progress (or lack of progress) toward achieving their goals. Reporting was perceived as either positive or negative depending on how well the goal had been defined and measured. A techno-timid participant shared her thoughts:

Having to report that I didn’t do what I was [supposed] to be doing made me think, “OK, well I’ll try to make that up on [another day] when I wasn’t supposed to exercise” ...So I think it was useful for me as a guilt thing sitting on my shoulder.

Female patient, 65-75 years

If patients found the goal meaningful and realistic, then the feedback from the tool was reinforcing, resonating well with the providers’ reflections on meaningfulness. However, sometimes setting goals incorrectly meant that they achieved the goal quickly; therefore, continuing use had little point. A techno-savvy participant shared his thoughts:

The goal was pretty simplistic and I set the goals probably too low. So it wasn’t really a challenge at all and I didn’t get a heck of a lot out of it.

Male patient, 75+ years

##### The Crucial Role of the Provider

The patients echoed the physician’s reflection that the collaborative nature of the goal-oriented approach was an important motivating factor. Both techno-savvy and techno-timid groups emphasized the importance of working with their health providers during the goal-setting process. Participants with positive experiences worked with their providers to identify relevant goals, and they negotiated meaningful levels of goal attainment. A patient added her suggestion:

Make sure the goal is stated in a way that is meaningful and can be tracked in a meaningful way. I would say the most important part of the whole thing for a patient would be working with the provider to get those goals just right.

Female patient, 45-54 years

The patient participants perceived the role of the provider in helping to narrow and focus the goal as encouraging and helped to identify areas that need work. For example, 1 techno-savvy participant described having set 3 goals. Progress was evident for 2 of the goals, but it became clear that more focused work was needed on the third goal. The participant independently came to this realization as she monitored her own progress on the tool:

I found the question related to how confident are you that you will reach your goal...was a really good question because as it went on, I could just see I went from I’m sure I can do this to Oh, it’s hopeless (laughs).

Female patient, 45-54 years

### Bringing It All Together to Generate a Content Theory

The narrative themes point to core mechanisms that are likely driving ePRO intervention outcomes. Most important are the notions of *meaningfulness* for patients and providers, which were influenced by key contexts including the patient-provider relationship (which enables collaborative goal setting), participant comfort with technology, the providers’ work environment, and the research process itself. These contexts moderate patient and provider adoption of the ePRO tool, which we expect will impact patient and provider outcomes. Most interesting is the role of the research process in relation to these contexts and how patients and providers assign meaning to intervention activities. For instance, stringent research timelines prevented more natural use of the tool in delivery of care, which forced participants to fit to the tool rather than incorporate it into their work and daily lives.

**Figure 2:**
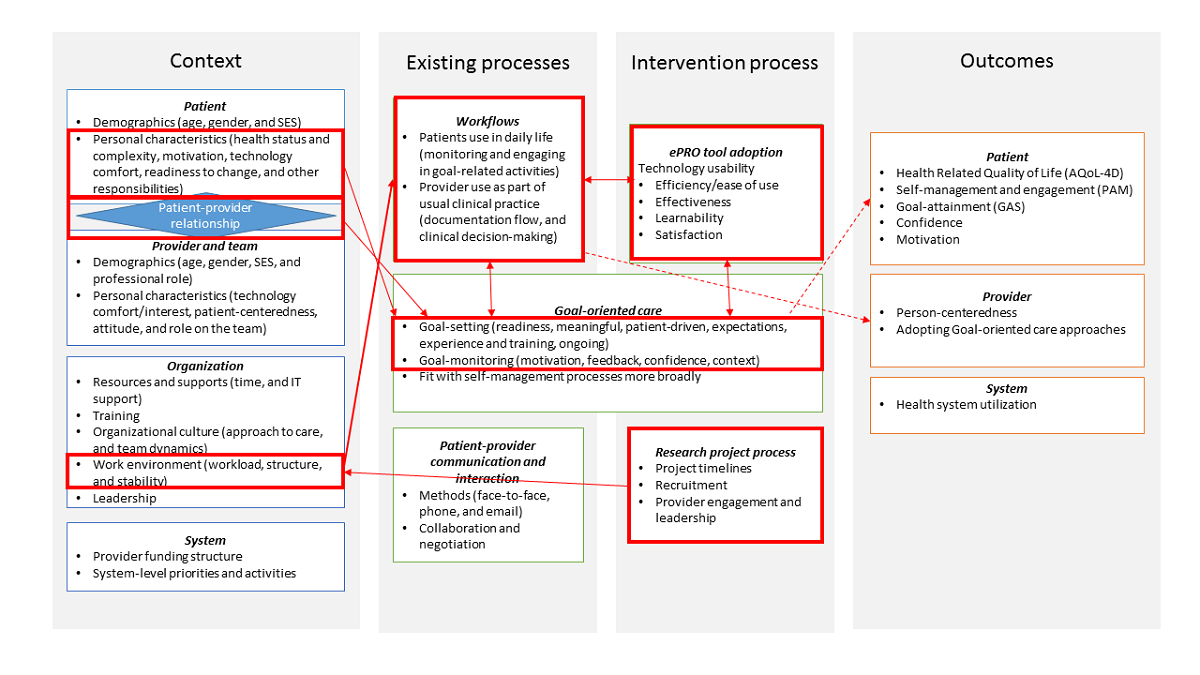
Dominant mechanisms driving electronic patient reported outcome trial outcomes. AQoL-4D: Assessment of Quality of Life Scale; PAM: Patient Activation Measure.

We offer a visual representation of how these mechanisms map onto our context, process, and outcome variables in Figure 2. Unidirectional arrows show how contexts and the research process influence the intervention process. Bidirectional arrows indicate processes that interrelate with each other over the course of the intervention. The dashed-line arrows indicate how we expect the processes to influence the outcomes collected at this stage, which would be tested in the next larger trial. Our discussion offers a likely theory to explain the nature of these mechanisms.

## Discussion

### Principal Findings

Outcome data alone suggest little change occurred for either control or intervention groups from pre to poststudy across any of our outcome measures. Intervention patients appeared to have a lower reported quality of life but higher levels of activation as compared with control groups. Notably, patient-centeredness seemed to go down for intervention patients according to the PACIC survey. Although the sample size is too low to capture real change, the narratives shed light on what variables might have an impact on these outcomes and point to areas that might be missed in looking at outcome data alone.

The narrative themes suggest fitting the ePRO tool into regular provider clinical work processes and patient daily activities is a critical mechanism and it points us in the direction of Normalization Process Theory (NPT) to uncover the nature of the mechanisms of our intervention. NPT’s emphasis on the social production and normalization of *work* helps us understand participant interactions with all aspects of the intervention, technological, processual, and social [46-48]. The theory suggests that new processes take hold through 4 generative mechanisms: coherence, cognitive participation, collective action, and reflexive monitoring. Through these mechanisms, new activities or practices become normalized and part of the everyday routine of actors.

We can see examples of all 4 generative mechanisms driving participant actions in the case of the ePRO tool. In terms of coherence (sense-making work), our narratives show how the perceived meaningfulness of the ePRO tool was crucial to overcoming contextual barriers (the research process) to adoption. Cognitive participation (relational work) and collective action (operational work) drove perceived meaning through patients and providers interacting with each other around the intervention, allowing them to legitimate tasks and assign roles and responsibilities when using ePRO. Engaging in these tasks effectively (setting up appropriate goals, patients remembering their goals and monitoring, and then following up together) was essential to meet outcomes reported in qualitative findings. When patients and providers saw the value of ePRO, through patients achieving and exceeding goals, meaningfulness increased and supported ongoing appraisal (or the reflexive monitoring mechanism) of the tool.

Many of these positive procedural changes are not captured in quantitative outcome data. The qualitative data provide a richer picture of outcomes that could be captured over the shorter term, such as patient motivation (related to empowerment), which can have a positive impact on outcomes [49]. Using narrative analysis, we are able to more clearly see the mechanisms of change needed to move the outcome dial over the longer term in a full-scale trial. For this trial we will need to do the following: (1) collect data on the perceived meaningfulness of ePRO to all participants (capturing coherence and relational work), (2) adopt a more pragmatic trial approach to better fit the research into the real-world environment (addressing operational work), and (3) pay careful attention to the process of goal-oriented care, particularly the patient and provider interactions and relationships (capturing relational work and reflexive monitoring). Critical to addressing all 3 aims is the adoption of an embedded ethnography that includes patient-provider interaction observations, interviews at multiple time points that probe on key areas, and an iterative analysis method that supports building interpretation of findings as we go. We will adopt Greenhalgh and Swinglehurst’s [29] approach to ethnographic information communication technology evaluation as a methodological and analytic guide.

### Limitations

One of the most important limitations in this study is that we did not explicitly aim to collect patient and provider narratives; therefore, our method had to be modified to look at shared stories on how participants used technology. One potential limitation is that we only conducted interviews with participants once, whereas narrative approaches often suggest multiple interviews to craft participant stories [36]. Instead of thorough additional interviews, we collected additional data regarding the stories of adopting ePRO through reflective memos and in team meetings. The team was engaged with the sites weekly, often daily, memoing on the exchanges and experiences, which allowed us to iteratively analyze and interpret the story of the intervention as we went. As noted previously, this type of iterative analysis is consistent with many qualitative method approaches including narrative [36].

Although the small sample meant we could not engage in more advanced statistical methods, it did allow us to do a much more in-depth qualitative analysis, which is perhaps more important at the stage of an exploratory trial. As noted in our findings section, the sample was also more homogenous than we expected, potentially reducing generalizability of findings to broader complex patient populations. However, it is possible that the goal-oriented care approach is more appropriate for a particular subset of patients with complex care needs; it is a new question we are exploring through another project in our current research program. However, the adoption of a theoretical framework and in-depth qualitative analysis does support transferability [50] of findings to other settings.

Finally, we were missing ethnographic observation, which potentially limited our ability to generate additional insights into the role of the patient and provider relationship and could have shed some light on some of the contradictions found in this study. Our full trial of the ePRO tool will include an embedded ethnography with more explicit use of narrative interview methods as a means to address these gaps.

### Conclusions

Evaluating complex interventions marks a significant methodological challenge, which may be especially crucial in interventions that incorporate technology as an added layer of social complexity. Simply identifying all likely contexts, processes and outcomes, and underlying mechanisms might be unwieldy, leading to more questions than answers with regard to findings from our studies. Furthermore, this might make it difficult or impossible to clearly identify the content theory driving the intervention. We suggest studies of complex interventions, particularly those that incorporate eHealth technologies, adopt phased and integrative evaluation methods as we have done here. Using narrative analysis as a part of exploratory trials offers a useful methodological approach to help identify more central mechanisms underlying our complex interventions that drive outcomes.

## Appendix

Multimedia Appendix 1 The electronic Patient Reported Outcome (ePRO) mobile app and portal screenshots.

## Abbreviations

AQoL-4D: Assessment of Quality of Life Scale
eHealth: electronic health
EMR: electronic medical record
ePRO: electronic patient-reported outcomes
FHT: Family Health Team
IT: information technology
NPT: Normalization Process Theory
PACIC: Patient Assessment of Chronic Illness Care
PAM: Patient Activation Measure
PSSUQ: Post-Study System Usability Questionnaire
